# Public Knowledge of Osteoarthritis in Al-Qunfudah Governorate, Saudi Arabia

**DOI:** 10.7759/cureus.34892

**Published:** 2023-02-12

**Authors:** Safa H Alkalash, Ali A Alsyed, Abdullah A Alrashdi, Ali S Alqarni, Mohammed A Alessa, Haitham H Alzubaidi, Ali H Alfaqih, Hassan R Alsuhabi, Ebrahem R Alsohabi

**Affiliations:** 1 Community Medicine and Health Care, Umm Al-Qura University, Al-Qunfudah, SAU; 2 Family Medicine, Menoufiya University, Shebin Alkom, EGY; 3 Medicine and Surgery, Umm Al-Qura University, Al-Qunfudah, SAU; 4 Mathematics, Umm Al-Qura University, Al-Qunfudah, SAU

**Keywords:** saudi arabia, osteoarthritis, knowledge, joint pain, arthritis

## Abstract

Background: Joint pain is one of the most frequent complaints among adults and older people in primary healthcare settings worldwide. There are many causes for joint pain, osteoarthritis (OA) is so far the most prevalent form of arthritis that causes joint pain. It can attack almost any joint, but the most frequently affected joints are the hands, knees, hips, and spine. This study aimed to identify public knowledge of OA and its associative variables in Al-Qunfudah governorate, Saudi Arabia.

Materials and methods: A cross-sectional descriptive community-based study was carried out among the general population in the Al-Qunfudah governorate. The research data were collected over two months, from November to December 2022, via an Arabic version of a self-administrated online survey of 29 items.

Results: A total of 746 respondents were included in this study. The majority of them were females (78%). The age group 18-29 was predominant. In terms of education, 69.9% were holding university degrees. The overall participants' knowledge of OA was poor at 36.1%, fair at 36.8%, and good at 26.9%. The associative variables with better participants' knowledge were; holding university degrees (P=0.021), being a student (P<0.001) and living in urban areas (P=0.020), having normal BMI (P=0.018), and depending on the school topics as a source of information (P<0.001). Good knowledge was significantly higher among healthy individuals and non-smokers (P<0.001) for each variable.

Conclusion: This study reveals the lack of knowledge of osteoarthritis among the general population in Al-Qunfudah governorate, Saudi Arabia. Being a student, university educated, from urban areas, and having a normal BMI, all were associative factors with good knowledge. Therefore, this study highlights the necessity for providing awareness and educational campaigns for the public, focusing on the rural population.

## Introduction

Osteoarthritis (OA) is a chronic bone disease and is considered the most common form of arthritis, with 240 million people affected worldwide [1]. OA is a progressive degenerative joint disorder that is characterized by cartilage damage, changes in the subchondral bone, osteophyte formation, muscle weakness, and inflammation of the synovium tissue and tendon. Although OA has long been viewed as a primary disorder of articular cartilage, the subchondral bone is attracting increasing attention [2]. The most commonly affected joints are the hands, knees, hips, and spine. It may be confined to one or a few joints of the affected patients [3]. Osteoarthritis occurs in two types; primary and secondary. The actual cause of articular degeneration in primary osteoarthritis is unknown, while secondary osteoarthritis is due to either abnormal force on the joint such as with post-traumatic causes, or abnormal articular cartilage, like rheumatoid arthritis [4].

Symptoms and signs of OA are usually pain, stiffness, swelling that limits joint movement, crepitus, restricted movement, bone enlargement, joint effusion, and bone instability [5]. Radiographic imaging can visualize bony features, including marginal osteophytes, subchondral sclerosis, and subchondral cysts that are associated with OA [6]. Magnetic resonance imaging (MRI) is the most beneficial procedure to accurately and feasibly measure the changes in quantitative cartilage morphometry for knee OA [7]. Management of osteoarthritis involves; non-pharmacological interventions like exercises, weight loss when appropriate, education, and physical therapy [1]. Swimming is the most beneficial form of exercise for patients suffering from OA and helps in relief of the disease-accompanied symptoms and joint stiffness in addition to improvement of muscle strength [8]. Pharmacological treatment; includes topical or oral NSAIDs provided that there is no contraindication for their usage in the affected patients. Intra-articular steroid injections are used to relieve pain for short-term patients with advanced symptoms and structural damage who are eligible for total joint replacement [1].

The global prevalence of knee OA is 16.0% in individuals aged 15 and over and 22⋅9% in individuals aged 40 and over [9]. A systematic review study detected OA of the knee was highly prevalent among women rather than men [10]. Moradi-Lakeh et al. stated that the range of point prevalence of osteoarthritis (per 1000) among the EMR countries was 9.7-37.3 [11]. Another cross-sectional study in KSA; looked at the radiographic evidence of osteoarthritis (OA) of the knee in 300 randomly chosen patients attending primary care facilities for different medical conditions. Radiographic OA was seen in males (53.3%) and females (60.9%) [12]. It is essential to increase population knowledge about highly prevalent disabling diseases like OA; this would improve their quality of life and adherence to treatment which, in turn, would improve the population’s experience with diseases and decrease healthcare costs.

Recently, in Saudi Arabia, a few studies revealed variable levels of knowledge about osteoarthritis among the Saudi population; some of them detected a lack of knowledge about OA [13,14]. While the others recorded an adequate level of public knowledge regarding OA [15]. On the international aspect, a study in Malaysia in 2014 suggested inadequate knowledge of OA [16]. There is no literature related to the public knowledge of OA in the Al-Qunfudah governorate. When we interviewed a sample of adults during our previous community awareness campaigns about osteoporosis disease, we noticed that many individuals in the Al-Qunfudah governorate confused osteoarthritis with osteoporosis and used both terms to describe any bone-related pains. Thus, this study was done to identify the awareness and associative variables of osteoarthritis among the population in the Al-Qunfudah governorate, Saudi Arabia.

## Materials and methods

Study design and setting

A cross-sectional descriptive community-based study was carried out among the general population in Al-Qunfudah governorate, Saudi Arabia, over six months, starting from August 2022 to January 2023. The study included males and females aged 18 years and more.

Al-Qunfudah governorate was the place where we conducted this study; it exists in the Tihamah plain on the Saudi Red Sea coast in Makkah Province and about 400 kilometers south of the Jeddah governorate. It represents about 3.7% of the regional area.

Study sample

The sample size was determined through the application of EPI Info.TM (CDC, Atlanta, GA) [17], depending on the overall population size in Al-Qunfudah governorate (300516), with a confidence interval (95%) and margin of error (5%). Finally, the calculated sample size was 384.

Tool and procedure for data collection

We have generated an online survey via the Google Document application, and this process has been done in many phases. Firstly, we conducted a focused literature review, followed by a selection of the relevant information, then the survey items were created and drafted as a 29-item Arabic survey. Its items were organized and reviewed by a panel consisting of three experts from different specialties (Family Medicine, Orthopedics, and Internal Medicine) who assessed the relevancy of each item to the research topic. Finally, it was pre-tested through the application of a pilot study. The main target of this pilot was to assess the relevancy of the designed survey before its use, to determine whether it would be understandable by the public with different educational levels and varied backgrounds, the time needed for its filling, and response rates. The survey link was disseminated on Al-Qunfudah Snapchat, and the public was asked to participate in this study voluntarily. The first 50 submitted answers were collected; then, the survey link was closed until the collected data have been analyzed. Finally, the reliability of the survey was evaluated by using the technique of test-retest. Its internal consistency was assessed, and Cronbach's Alpha coefficient test was (0.83). The final form of the applied survey was composed of 29 questions and distributed into two sections. The initial part involved 12 questions about demographics and clinical information of the study group, such as age, gender, residence, educational level, employment, weight, height, body mass index and their history of OA, joint surgeries, daily exercise, and smoking status. The second section involved 17 questions that inquired about their knowledge of osteoarthritis, such as its definition, causes, prevalence, affected joints, the difference between it and osteoporosis, its clinical manifestations, complications, and management.

The main study data were collected over two months, from November to December 2022, through the predesigned survey that was distributed on different electronic applications such as WhatsApp, Twitter, Snapchat of Al-Qunfudah, and Facebook. To ensure that all participants were from the Al-Qunfudah governorate, the survey was disseminated on Al-Qunfudah Snapchat. As well it involved a question about the residence of each participant, and it was planned to discard any response from outside Al-Qunfudah governorate, but when we reviewed the collected data, we found that all respondents were from it, so we did not exclude any responses. Finally, the total number of completed questionnaires was 726. Therefore, the study sample was achieved. There were no incomplete questions because the questionnaire was designed in the required answering manner.

Scoring of knowledge

A good knowledge score of osteoporosis was estimated when the participant could correctly answer 80% or more of the questions, a fair knowledge score for whom correctly answered 79%-60%, while less than 60% was considered a poor knowledge score [18].

Ethical considerations

This study received an ethical approval number (HAPO-02-K-012-2022-11-1289) from the Medical Research and Ethical Committee of the College of Medicine, Umm Al-Qura University, Makkah, KSA. The collected data are confidential, and consent was obtained from each participant through an introductory question at the beginning of the applied survey.

Statistical analysis

The data were processed before the statistical analysis. Data management and analysis were carried out by using the IBM Corp. Released 2012. IBM SPSS Statistics for Windows, Version 21.0. Armonk, NY: IBM Corp. Quantitative data were expressed as means and standard deviations, while qualitative data were expressed as numbers and percentages. We compared participants’ awareness of osteoarthritis and its related factors among demographic groups using the Chi-squared test. A statistical significance was considered when P < 0.05.

## Results

A total of 746 completed questionnaires. The majority of the participants (78%) were females, and (79.3%) were living in Al-Qunfudah city. Two-thirds of the study subjects belonged to the age group of 18-29 years. In terms of education, (69.9%) had university degrees. The mean weight is 60 kg, and the mean height is 160 cm; approximately 87% and 91% of them had never been diagnosed with OA or undergone any surgical operations in their joints (Table 1).

**Table 1:** Distribution of the study group according to their demographics and clinical characteristics (n=746) All values are presented as numbers (n) and percentages (%). Mean ± Standard deviation (M±SD). Kilograms (kg). Centimeters (cm). Kilogram/squared meter (kg/m2). Osteoarthritis (OA).

Variables		n	%
Sex	Female	582	78.02%
	Male	164	21.98%
Residence	Al-Qunfudhah city	591	79.22%
	Al-Qunfudhah related villages	155	20.78%
Age	18-29	493	66.09%
	30-39	121	16.22%
	40-49	85	11.39%
	50 and above	47	6.30%
Education	Secondary	205	27.48%
	University	522	69.97%
	Post-graduate	16	2.14%
	Intermediate and below	3	0.40%
Occupation	Government employee	164	21.9%
	Non-governmental employee	98	13.1%
	Student	206	27.6%
	Housewife	151	20.2%
	Retired	27	3.6%
	Non-working	100	13.4%
Weight	21-162 kg (60.04 ± 16.3)		
Minimum-maximum (M±SD)			
Height	120-180 cm (160.0±8.8)		
Minimum-maximum (M ±SD)			
Body mass index (BMI)	Underweight (Less than 18.5 kg/m^2^)	142	19.0%
	Normal (18.5-24.9 kg/m^2^)	360	48.3%
	Overweight (25.0-29.9 kg/m^2^)	163	21.8%
	Obese (30.0-39.9 kg/m^2^)	75	10.1%
	Morbid obese (40.0 and more kg/m^2^)	6	0.8%
Self-reported OA	Yes	96	12.9%
	No	650	87.1%
Previous joint surgery	Yes	66	8.8%
	No	680	91.2%
Daily exercise	Yes	249	33.4%
	No	497	66.6%
Smoking status	Smokers	93	12.5%
	Non-smokers	653	87.5%

About 91% of the study subjects indicated that osteoarthritis is not a synonym for osteoporosis. Half of them correctly identified the underlying mechanism of OA as a process during which the joint cartilage begins to wear out over time. The definition of osteoporosis was well-known by 82.4% of them, who reported that it reflects bone fragility and easiness to be broken in osteoporosis. Approximately half of the respondents (49.6%) stated that osteoarthritis is a chronic disease, and (74.5%) stated that it is a common disease. About half (55%) were able to recognize that joint pain is the most common but not the solitary symptom in OA, 56 % also identified that OA causes joint stiffness, and 62% reported that swelling is a common sign in OA. More than two-thirds of the study group (71%) gave a consensus on OA could lead to loss of joint movement (Table 2).

**Table 2:** Distribution of the study sample according to their response to knowledge items of osteoarthritis (n=746) All values are presented as numbers (n) and percentages (%). Osteoarthritis (OA).

Items	Correct Answers	
	n	%
There is a difference between joint osteoarthritis and osteoporosis	680	91.15%
The cause of the joint osteoarthritis	404	54.16%
Definition of osteoporosis	615	82.44%
Osteoarthritis is a chronic disease	370	49.60%
Osteoarthritis is a common disease	556	74.53%
There are certain joints that are greatly affected	608	81.50%
The most affected joints of joint osteoarthritis	515	69.03%
Infection with micro-organism is related to the disease of OA	234	31.37%
Pain is the only presentation of the disease	412	55.23%
Sclerosis is shown to the osteoarthritis of the joints	420	56.30%
Joint swelling (swelling) is a sign of OA	465	62.33%
Osteoarthritis may lead to lose of joint movement	532	71.31%
Genetic factors can predispose to osteoarthritis	391	52.41%
Age is a factor causing joint osteoarthritis	627	84.05%
The rate of OA in men and women are equal in the osteoarthritis	369	49.46%
X-rays are used to diagnose OA	336	45.04%
Medications such as aspirin, for example, improve symptoms	216	28.95%

**Figure 1:**
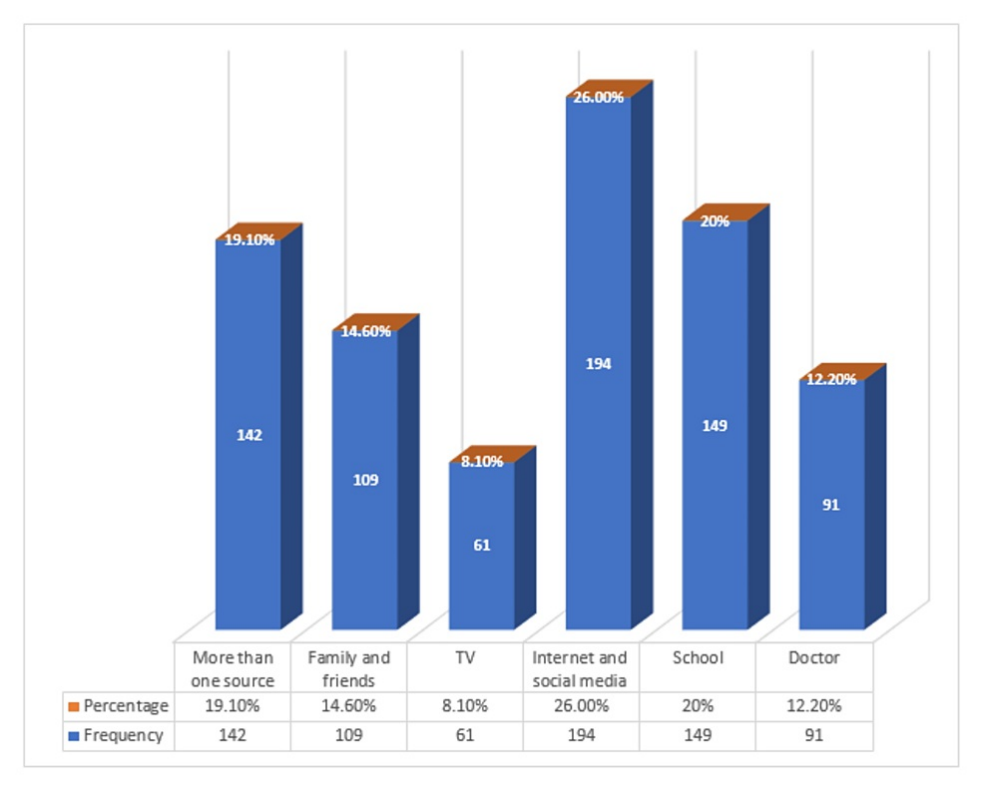
Source of knowledge of osteoarthritis among the general population in Al-Qunfudah governorate

Internet and social media were the most common sources of information (26%) about OA among this study's participants (Figure 1).

**Figure 2:**
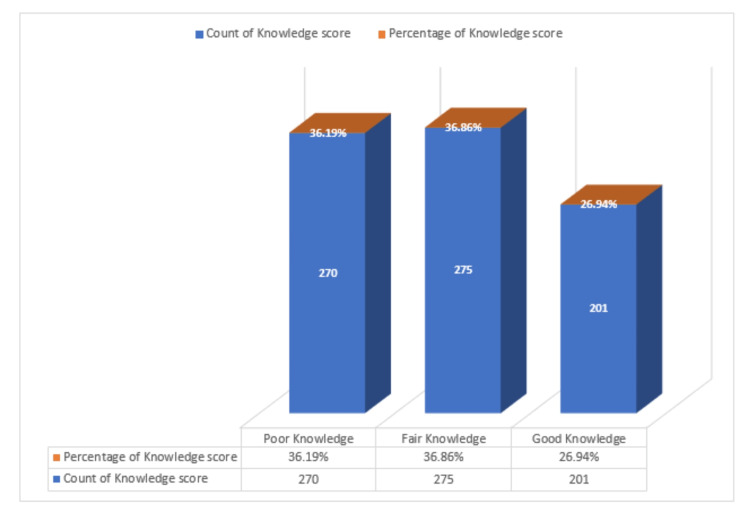
Participants' knowledge score of osteoarthritis (n=746)

The overall participants' knowledge of OA was poor at 36.1%, fair at 36.8%, and good at 26.9% (Figure 2).

On relating overall knowledge level with the participants' sociodemographic characteristics, 82% of those who were living in Al-Qunfudah city had recorded better knowledge levels than those living in the surrounding villages, with a significant difference found (P=0.020). Furthermore, 79% of university-educated participants had better knowledge scores (P=0.021). Good knowledge of OA was observed among students and those who obtained their information during learning in school with (P <0.001) for each. Near a third of those having poor knowledge were overweight (P= 0.018) (Table 3).

**Table 3:** Relationship between participants' knowledge scores of osteoarthritis and their demographic characteristics (n=746) All values are presented as numbers (n) and percentages (%). Osteoarthritis (OA). Kilogram/squared meter (kg/m2). Statistically significant (P value<0.05).

Variables		Knowledge score			P-value
		Poor, n(%)	Fair, n(%)	Good, n(%)	
Age in years	18-29	169 (62.6%)	183 (66.6%)	141 (70.2%)	0.321
	30-39	53 (16.6%)	44 (16.0%)	24 (11.9%)	
	40-49	33 (12.2%)	32 (11.6%)	20 (9.9%)	
	More than 50	15 (5.6%)	16 (5.8%)	16 (7.9%)	
Sex	Female	201 (74.4%)	213 (77.5%)	168(83.6%)	0.062
	Male	69 (25.6%)	62 (22.6%)	33 (16.4%)	
Residence	Al-Qunfudah city	223(82.6%)	203 (73.8%)	165 (82.1%)	0.020
	Al-Qunfudah-related villages	47 (17.4%)	72 (26.2%)	36 (17.9%)	
Education	Secondary	81 (30.0%)	88 (32.0%)	36 (17.9%)	0.021
	University	182 (67.4%)	180 (65.5%)	160 (79.6%)	
	Post-graduate	5 (1.6%)	6 (2.2%)	5 (2.5%)	
	Intermediate and below	2 (0.7%)	1 (0.4%)	0 (0.0%)	
Occupation	Governmental employee	65 (24.1%)	59(21.5%)	40(19.9%)	<0.001
	Non-governmental employee	43(15.9%)	35(12.7%)	20(9.9%)	
	Student	52(19.2%)	81(29.5%)	73(36.3%)	
	Housewife	44(16.3%)	62(22.5%)	45(22.4%)	
	Retired	14(5.3%)	7(2.5%)	6(2.9%)	
	Non-working	52(19.2%)	31(11.3%)	17(8.5%)	
Source of information	Doctors	21(7.8%)	31(11.3%)	39(19.4%)	<0.001
	School	45(16.7%)	49(17.8%)	55(27.3%)	
	Internet and social media	80(29.6%)	73(26.5%)	41(20.4%)	
	TV	20(7.4%)	20(7.3%)	21(10.4%)	
	Family and friends	46(17.0%)	37(13.5%)	26(12.9%)	
	Mixed sources	58(21.5%)	65(23.6%)	19(9.5%)	
Body mass index	Underweight (Less than 18.5 kg/m^2^)	45(16.6%)	49(17.8%)	48(23.9%)	0.018
	Normal (18.5-24.9 kg/m^2^)	115(45.6%)	154(56.0%)	91(45.3%)	
	Overweight (25.0-29.9 kg/m^2^)	73(27.0%)	49(17.8%)	41(20.4%)	
	Obese (30.0-39.9 kg/m^2^)	34(12.6%)	21(7.6%)	20(9.9%)	
	Morbid obese (40.0 and more kg/m^2^)	3(1.1%)	2(0.7%)	1(0.5%)	

On the other connection between overall knowledge of osteoarthritis with the participants' clinical and general information, participants who were not previously diagnosed with OA (94.5%) or had undergone any surgical operation in their joints (98.5%) had significantly better knowledge of OA (P <0.001). Additionally, better knowledge was highly detected among non-smokers (95.5%) (P <0.001) (Table 4).

**Table 4:** Relationship between participants' knowledge scores of osteoarthritis and their clinical and general information (n=746) All values are presented as numbers (n) and percentages (%). Osteoarthritis (OA). Statistically significant (P value<0.05).

	Knowledge score			P-value
	Poor, n (%)	Fair, n (%)	Good, n (%)	
Self-reported OA				<0.001
Yes	44 (16.3%)	41 (14.9%)	11 (5.5%)	
No	226 (83.7%)	234 (85.1%)	190 (94.5%)	
Previous joint surgery				<0.001
Yes	33 (12.2%)	30 (10.9%)	3 (1.5%)	
No	237 (87.8%)	245 (89.1%)	198 (98.5%)	
Daily exercise				0.161
Yes	91 (33.7%)	101 (36.7%)	57 (28.4%)	
No	179(66.3%)	174 (63.3%)	144 (71.6%)	
Smoking status				<0.001
Smokers	48 (17.8%)	35 (12.7%)	10 (5.0%)	
Non-smokers	222 (82.2%)	240 (87.3%)	191 (95.5%)	

## Discussion

Osteoarthritis (OA) is the most common disease and cause of disability in the elderly [19]. Therefore, it is crucial to raise awareness of osteoarthritis in the Kingdom of Saudi Arabia to help people adopt healthy habits and accept newly suggested prevention measures. This study examined how well-informed a proportion of the Al-Qunfudah population of Saudi Arabia identify knowledge of OA and its associative variables. Women made up 78% of the participants, and the population living in Al-Qunfudah city constituted (79.2%) of the sample. Only 6% of the participants were 50 years of age or older, while college students represented 69.9%. A total of 96 respondents (12.3%) self-reported having been diagnosed with osteoarthritis, and this was not shocking as previous literature reported that the overall prevalence of osteoarthritis in Saudi Arabia is 15.3% [12].

The majority of study participants (91%) have differentiated between osteoarthritis and osteoporosis, and about half of them have correctly identified the basic mechanism for OA (82.4%) and were aware that osteoporosis makes bones brittle and easily broken. Nearly half of the respondents (49.6%) have said osteoarthritis is a chronic condition, and 74.5% have stated that it is a common disease. These findings are much better than that from a previous study in Malaysia which revealed that 51.9% of its study subjects had known that osteoporosis is different from osteoarthritis [18]. The current higher percentage of those who could distinguish between osteoporosis and osteoarthritis is an outstanding finding because this may facilitate the prevention of both common diseases and their complications. 

The chief symptom of osteoarthritis is pain which usually warrants a doctor's visit. Stiffness is another symptom that usually goes away after 20-30 minutes, especially in the morning or after a period of inactivity [5]. The finding from this study went in the same direction as the previously mentioned fact, as about half of the respondents correctly answered that pain and stiffness are the main symptoms of OA. Alyami et al. reported the same percentage in their previous study in Jeddah [13]. Pain and stiffness of joints are very distressing symptoms that may interfere with the usual activity of the affected patients and constitute a bad experience that could not go unrecognized by them. The disease often begins with joint swelling (62%), and patients with OA suffer from restricted mobilization (71%). Participants (84%) were aware that age is a risk factor for developing OA. The same finding was reported in a Pakistani study which revealed that 63% of the studied group attributed to age as one of the risk factors for joint pain [20].

A previous systematic review by Tschon et al. concluded that women are more likely to develop OA and suffer more severely than men as a most observational cohort and cross-sectional designed studies which globally analyzed 268,956 patients, of which 103,700 were men (39%) and 165,256 women patients (61%) [21]. Unfortunately, only half of the participants were aware of this information. A well-known risk factor for OA is its high genetic component [22]. The estimate of heritability has been reported to be 40% for the knee, 60% for the hip, 65% for the hand, and about 70% for the spine [22]. This tip has been known by 52% of the sample. The failure of half of the study group to recognize the gender and genetic predisposition of OA is worrisome. Therefore, this knowledge should be highlighted in the upcoming educational campaigns for public orientation.

The diagnosis of OA can be made with just a simple film X-ray, history, and physical exam. It is rare to diagnose OA using biochemical markers in the blood [23]. The respondents' knowledge of using an X-ray and physical exam to diagnose OA has been recognized by only 45% of the participants. Regarding the treatment of OA, guidelines [23-25] recommend non-steroidal anti-inflammatory drugs (NSAIDs) as the first-line therapy and its action through inhibiting the production of prostaglandins (PG) and thromboxane A through the blockade of cyclooxygenase (COX). Currently, less than a third (28%) of participants were aware of the important role NSAIDs play in managing symptoms. This percentage is less than findings from another Saudi study which reported that 46% of the general population could know that NSAIDs are the drug of choice for treating symptoms of osteoarthritis [13].

The overall participants' knowledge of OA in this study was poor at 36.1%, fair at 36.8%, and good at 26.9%. In contrast with a previous study, which included 1052 participants from the Aseer region, the level of knowledge of OA was high, as 82.6% of the population had a good awareness level regarding OA in total [15]. Despite both studies being done in the kingdom of Saudi Arabia, there is a great discrepancy in their outcomes; the cause may be related to the difference in characteristics of both studies' participants; and the availability of health education campaigns as Al-Qunfudah governorate is a remote area with a limited number of health care settings when compared with Aseer region. Moreover, the difference in tools for data collection may be another cause of this dissimilarity between both studies' findings.

According to the current study, 70% of participants belonging to the age group of 18-29 years and university-educated respondents have shown better knowledge levels than the others. This result is in disagreement with both Saudi and Malaysian studies, which concluded that participants aged 50 and up had a higher level of knowledge and awareness about OA [15,16]. The dissimilarity in these studies' results may be due to the differences in the study settings and time. Nowadays, young people have numerous access to getting information much easier than in previous times due to the availability of the internet, social media, and modern study curricula. Being an educated person will facilitate searching for information. A significant difference has been found between residence and good knowledge of OA, as participants from the urban area have shown better knowledge. This could be due to the more opportunity for public awareness campaigns and accessibility of better internet connections in urban rather than rural areas.

In recent years, there is a great change in the schools' educational process and students' curricula that provide students with a large stock of evidence-based knowledge concerned with health. Thus, it was an unsurprising issue to identify better knowledge of OA among the study participants who took their information from the school. On the other side, poor knowledge was detected clearly among those who depended on social media in getting their health-related information. The main cause of this outcome is that persons who provide information on social media may be unprofessional. Therefore, their information is not evidence-based, is poor quality, and carries the risk of being fake. This finding should be considered by policymakers to establish measures to ensure the availability of evidence-based information on social media, as it is the most popular and accessible method of public education. A few years ago, Tonsaker et al. warned that the distribution of poor-quality information can harm patients and damage the professional image. There is a high risk of misinformation, as healthcare providers are unable to control the content that is posted or discussed to a previous study finding [21].

It was known that obese individuals have a higher risk for OA, with every 5 kg of weight gain increasing the risk of knee OA [26]. There is evidence that the risk accumulates with high BMI throughout adulthood, with an association between BMI and later knee OA starting as early as 20 years in men and 11 years in women [26. This study noted poor knowledge of OA in a third of overweight people; therefore, health education sessions should be directed to the public to explore the risk of obesity on the joint. Additionally, this study reveals that good knowledge was obvious among participants who had never been diagnosed with OA or undergone surgical operations in their joints, and this result against that was detected in a previous study which reported that individuals who were previously diagnosed with OA had significantly higher knowledge than others who were not [27].

Previous literature concluded that exercise has positive salutary benefits for joint tissues in addition to its other health benefits [5].In this study, the majority of participants who practiced exercise daily possessed a fair knowledge level of OA (28.36%), and this finding is in disagreement with what was reported by Hussain and Abdul Raheem in their study, which revealed that physical activity showed no statistically significant change to the odds of having good awareness [28]. Moreover, evidence suggests that cigarette smoking may have a negative effect on cartilage metabolism [29]. In the current study, a significant association was found between knowledge and smoking status among the study subjects, as good knowledge was mostly detected in non-smokers (95.5%). This outcome is reassuring as it may be due to the recognition of the bad effects of smoking on the joint and cartilage which motivated these participants to avoid smoking and maintain their joint health.

The limitations of the study. First, the online self-administered questionnaire may have the disadvantage of applying only to those who can read, familiar to use the internet technologies, and have internet connections. Therefore, the study has denied the awareness level of osteoarthritis in a respected sector of the community, which may involve older people who are at higher risk for OA and its consequences. Second, more than two-thirds of the study sample was from Al-Qunfudah city, which is a relatively urban region; thus, the knowledge scores reflected mainly awareness of the urban population with deprivation of those from rural regions. Despite the previously mentioned limitations, this study is an initiative to highlight this common disease in the general population.

## Conclusions

This study reveals the inadequate level of knowledge among the general population in Al-Qunfudhah Governorate, Saudi Arabia, about osteoarthritis. University education, still studying, urban residence, normal body mass index, and being a non-smoker; all were associated variables with good knowledge. Awareness campaigns are highly recommended to educate the public properly through simplified evidence-based information about the such debilitating disease. Collaboration between the healthcare sector and other community organizations is a vital step to expanding educational programs in the community. Social media should be monitored by policymakers and health organizations to limit the rapid spreading of unprofessional health-related information and provide alternatives of short health educational messages in simple language to be feasible for all general population regardless of their education or background.
